# CD24 Expression Is Increased in 5-Fluorouracil-Treated Esophageal Adenocarcinoma Cells

**DOI:** 10.3389/fphar.2017.00321

**Published:** 2017-05-30

**Authors:** Pilar Jiménez, Eduardo Chueca, María Arruebo, Mark Strunk, Estela Solanas, Trinidad Serrano, María A. García-González, Ángel Lanas

**Affiliations:** ^1^CIBERehdMadrid, Spain; ^2^Instituto de Investigación Sanitaria Aragón (IIS Aragón)Zaragoza, Spain; ^3^Centro de Investigación Biomédica de Aragón, IACS Aragón, Instituto Aragonés de Ciencias de la Salud, Servicio de Secuenciación y Genómica FuncionalZaragoza, Spain; ^4^Department of Gastroenterology, Hospital Clínico Universitario Lozano BlesaZaragoza, Spain; ^5^Instituto Aragonés de Ciencias de la Salud (IACS)Zaragoza, Spain; ^6^Department of Medicine, University of ZaragozaZaragoza, Spain

**Keywords:** esophageal adenocarcinoma, cancer stem cells, CD24, celecoxib, 5-fluorouracil

## Abstract

The cancer stem cell (CSC) model suggests that there are subsets of cells within a tumor with increased proliferation and self-renewal capacity, which play a key role in therapeutic resistance. The importance of cyclooxygenase-2 (*COX-2*) in carcinogenesis has been previously established and the use of *COX-2* inhibitors as celecoxib has been shown to exert antitumor effects. The present study investigated whether treatment of esophageal adenocarcinoma (EAC) cells with 5-fluorouracil (5-FU) or the growth of tumor spheres increased the proportion of CSCs and also if treatment with celecoxib was able to reduce the putative CSC markers in this tumor. OE19 and OE33 EAC cells surviving 5-FU exposure exhibited an increase in CSC markers *CD24* and *ABCG2* and also an increased resistance to apoptosis. EAC cell lines had the capacity to form multiple spheres displaying typical CSC functionalities such as self-renewal and increased *CD24* levels. In addition, after the induction of differentiation, cancer cells reached levels of *CD24* similar to those observed in the parental cells. Treatment with celecoxib alone or in combination with 5-FU also resulted in a reduction of *CD24* expression. Moreover, celecoxib inhibited the growth of tumor spheres. These findings showing a reduction in CSC markers induced by celecoxib suggest that the *COX-2* inhibitor might be a candidate for combined chemotherapy in the treatment of EAC. However, additional clinical and experimental studies are needed.

## Introduction

The incidence of EAC has increased dramatically in the last decade in western countries. This tumor has a high index of radio- and chemo-resistance, and is frequently diagnosed at an advanced state; therefore, EAC has poor prognosis with a 5-year survival rate ranging from 15 to 39% despite the use of combined therapies (van Lanschot et al., 2002; DeMeester, 2006).

The limited effectiveness of standard anticancer therapies has been attributed to the presence of cancer stem cells. The CSC model proposes that tumors are organized hierarchically, with a subpopulation of stem-like cells which is responsible for sustaining tumor growth. These CSCs have the capacity to self-renew and differentiate to generate the cellular heterogeneity observed in tumors, and may play a pivotal role in local invasion, metastasis, resistance to therapy, and subsequent tumor recurrence. Cancer stem cells share many of the properties of normal stem cells, including relative quiescence, an active DNA-repair capacity, and resistance to apoptosis (Reya et al., 2001; Dean et al., 2005; Dallas et al., 2009; Yoon et al., 2014). Previous reports have demonstrated that chemoresistant cells are enriched for CSC markers and that chemotherapy can lead to the propagation of CSCs and prevent their differentiation (Levina et al., 2008; Dallas et al., 2009; Abubaker et al., 2013; Canter et al., 2014). Thus, the identification of CSCs is important because it might provide new targets for cancer therapy and may be used to develop a new class of biomarkers.

Experimental and clinical studies have demonstrated the existence of CSCs in a wide range of hematopoietic and solid tumors including colon, breast, prostate, pancreas, brain, and liver (Al-Hajj et al., 2003; Singh et al., 2003; Kristiansen et al., 2004a; Haraguchi et al., 2005; Li et al., 2007; Yu et al., 2009; Zhang et al., 2010; Cao et al., 2011; Zhao et al., 2012; Liu et al., 2013). These cells are characterized by a distinctive profile of surface markers, their ability to form spheres of self-replicating cells *in vitro*, and the capacity to form tumors in immunodeficient mice (Reya et al., 2001; Rosen and Jordan, 2009).

To date, definitive cell surface immunophenotypes have not been defined for most of CSCs and knowledge about CSCs in EAC is very limited. Previous reports identified a side population in EAC cell lines, and *CD34* was reported as a potential stem cell marker in the mouse esophagus (Haraguchi et al., 2005; Kalabis et al., 2008; von Rahden et al., 2011; Zhang et al., 2012; Zhao et al., 2012). Studies in human EAC tissues identified a tumor-initiating stem-like subpopulation of cells which did not express any of the common cell surface markers identified as CSC markers in other types of cancer (Grotenhuis et al., 2010).

*COX-2* are membrane proteins that catalyze prostaglandins production. *COX-2* overexpression is related to the development of GI cancers, and epidemiological studies have shown that nonsteroidal anti-inflammatory drugs (NSAIDs) exert chemopreventive effects on EAC (Farrow et al., 1998; Anderson et al., 2006; Abnet et al., 2009). Celecoxib, a *COX-2* specific inhibitor, has also been tested as a chemotherapeutic agent, decreasing the neoplastic aggressiveness of esophageal adenocarcinoma when used as neoadjuvant therapy (Tuynman et al., 2005). Nowadays there are clinical reports of the effectiveness of combining selective *COX-2* inhibitors with chemotherapy to treat digestive tract tumors, but the exact mechanism underlying the anti-tumor effects remain unclear (Dawson et al., 2007; Altorki et al., 2011).

Given the relationship between chemoresistance and the CSC phenotype, our first approach was to analyze whether esophageal cancer cells that survived drug treatment were enriched in CSC markers (previously established as CSC markers in other human cancers), and to investigate the CSC phenotype in esophageal spheres from cancer cell lines. Finally, we investigated if celecoxib could be related on the suppression of those markers in chemotherapy-induced CSCs.

## Materials and methods

### Cell lines and culture conditions

The EAC cell lines (OE19 and OE33) were derived from human EAC and were purchased from the European Collection of Cell Cultures (ECACC; Sigma, St. Louis, MO). The OE33 cell line was established from an adenocarcinoma of the lower esophagus arising in Barrett's esophagus and exhibited poor differentiation. The OE19 cell line was established from an adenocarcinoma of gastric cardia/esophageal gastric junction and exhibited moderate differentiation.

Cells were cultured in RPMI 1640 medium supplemented with 2 mM glutamine containing 10% fetal bovine serum (FBS) and antibiotics (100 U/mL penicillin G, 100 μg/mL streptomycin, and 0.25 μg/mL amphotericin) in a humidified atmosphere of 5% CO2/95% air at 37°C.

### MTT assay

The effect of 5-FU (Sigma) treatment on cell viability was evaluated by MTT. Briefly, EAC cells were seeded in 96-well-plates at a density of 2,500 cells/well in 200 μL of medium. After seeding, cells were incubated overnight. The following day, cells were treated with different concentrations of 5-FU (1, 10, 50, or 100 μg/mL), and then incubated for 72 h. Next, cells were washed and treated with MTT for at least 2 h. Colorimetric analysis was performed at a wavelength of 570 nm using a standard microplate reader. To determine cell viability, percent viability was calculated as [(absorbance of drug-treated) sample/(control absorbance)] × 100. 5-FU was dissolved in DMSO as a stock solution. All the assays were performed in triplicates, in three independent experiments.

### RNA extraction and gene expression analysis

Cells were grown in culture in 175-cm^2^ flasks until they reached 70–80% confluence. Then, cells were treated with 5-FU at IC_50_ concentration (10 μg/mL). After 72 h of treatment, cells were rinsed with PBS, and the surviving cells were subjected to RNA extraction using an RNeasy Fibrous Tissue Kit (Qiagen, Crawley, Surrey, UK) according to the manufacturer's instructions. The total RNA isolated was purified using RNeasy Mini Elute Cleanup (Qiagen) and quantified by spectrophotometry.

Relative gene expression was determined using the GeXP genetic analysis system (Beckman Coulter, Barcelona, Spain), which allows multiplex detection and quantitation of gene sets in a single analysis (Rai et al., 2009). RT reactions (10 μL) contained 50 ng RNA, 200 nM reverse primers, 2.5 μL kanamycin resistant (Kan^r^) RNA, 2 μL 5X RT Master Mix buffer, and 0.5 μL reverse transcriptase. The conditions of RT reactions were: 1 min at 48°C, 5 min at 37°C, 60 min at 42°C, and 5 min at 95°C. Reverse transcriptase, RT master mix buffer, and Kan^r^ RNA were supplied in Genome Lab GeXP Start Kit. Intron spanning primers were designed using the GenomeLab eXpress Designer software and expression analysis was done with the GenomeLab eXpress Profiling software (Beckman Coulter). Multiplex amplified fragments were separated on a GenomeLab XP capillary sequencer and peak areas were normalized using a reference gene to calculate relative gene expression.

The PCR reaction was performed using 4.65 μL of the 10 μL RT reaction and a final concentration of 200 nM forward primer. 25 mM MgCl_2_, 5X PCR buffer, and ThermoTaq polymerase were also added at the concentrations indicated in the Genome Lab GeXP Start Kit. The PCR reaction conditions were: 10 min at 95°C; followed by 35 cycles of 30 s at 94°C, 30 s at 55°C, and 1 min at 68°C. PCR products were analyzed on the GeXP after dilution (800x) with sample loading solution. Kanamycin was used as the RNA internal positive control.

We determined the expression of *CD24, CD34, CD44, CD133, ESA, ABCG2, BAX, BCL2L1*, and *LGR5* genes. For relative gene expression calculation, raw data for each gene were normalized to the housekeeping gene *TBP* and were subsequently calculated relative to an internal standard (*Kan*^*r*^ gene). The primers are summarized in Table 1. All the experiments were performed in triplicate.

**Table 1:** PCR primers for multiplex-PCR.

**Gene/symbol**	**Accession N°**	**Primer Forward (5′–3′)**	**Primer Reverse (5′–3′)**	**Size (bp)**
CD24	NM_013230	GAGACCACGAAGAGACTGGC	ACCCACGCAGATTTATTCCA	156
CD34	NM_001773	CTGGAGTTGAAACGTTGGCT	GGCCACAACAAACATCACAG	149
CD44	NM_001001392	CAGGTCTCAAATCCGATGCT	TACAGCATCTCTCGGACGG	107
CD133	NM_006017	GGCTAGTTTTCACGCTGGTC	ACCGACTGAGACCCAACATC	165
ESA	NM_004475	GCTGCACAACCTCAATCTCA	TCAGCCTTCAGTGAGGAGGT	121
ABCG2	NM_004827	AAGCCATTGGTGTTTCCTTG	TGAGCCTTTGGTTAAGACCG	114
BAX	NM_004324	GCCACTCGGAAAAAGACCTC	CAGCTCTGAGCAGATCATGAAG	246
BCL2L1 (BCL2-like1)	NM_138578.1	GTATCACAGGTCGGGAGAGG	CAATTCCTGTGTCGCCTTCT	142
Lgr5	NM_003667	GCAGACGGTTTGAGGAAGAG	CCCCACTGCAATTAGGACAC	298
TBP	NM_003194	GGAGGCAAGGGTACATGAGA	CCCCACTGCAATTAGGACAC	268

### Immunofluorescence staining of cells

Cells were grown on sterile coverslips placed in 24-well-plates. After 72 h, cells were washed and fixed in methanol for 15 min at −20°C. Cells were then incubated with the primary *CD24* antibody (diluted 1/100 in PBS/1% BSA; clone 32D12, Dianova, Hamburg, Germany) or *ESA* antibody (diluted 1/200; Abcam, MA USA) for 90 min at room temperature in a high-humidity chamber. After washing twice with PBS, the Alexa Fluor 488 goat anti-mouse (1/250 dilution) and Alexa Fluor 488 goat anti-rabbit (1/250 dilution) were added, respectively, and cells were incubated at room temperature for 1 h in a high-humidity chamber.

After several washes, coverglasses were mounted on glass slides using Mowiol. Preparations were observed in a fluorescence microscope (Olympus IX81, Tokyo, Japan).

### Flow cytometry assay

Control and 5-FU-treated (10 μg/mL) EAC cells were subjected to flow cytometry analyses to determine *CD24* and *ESA* levels. Briefly, the cells were harvested, centrifuged, and suspended in 50 μl PBS. Cells were then incubated with 5 μl mouse anti-human CD24-PE antibody (clone ALB9; Immunotech, Marseille, France) or ESA-FITC antibody (clone ESA214; GeneTex, Irvine, CA) for 15 min at 4°C. Mouse anti-human IgG1-PE and mouse anti-human IgG1-FITC were used as isotype controls for *CD24* and *ESA*, respectively. Samples were analyzed with a FACSAria cell sorter (BD Biosciences, San Jose, CA, USA).

Similarly, tumor spheres and cells treated with celecoxib were subjected to flow cytometric analysis to examine *CD24* expression. All the experiments were performed at least three times.

### Sphere formation assay

Tumor sphere cultures were established from trypsinized cells from adherent cultures of the parental OE33 and OE19 cell lines. For the formation of esophageal spheres, cells were plated in 25 cm^2^ ultra-low attachment flasks (Corning, Tewksbury, MA, USA) and cultured in serum-free Dulbecco's modified eagle medium (DMEM-F12, Sigma) containing: 5 μg/mL insuline (Sigma), 0.4% bovine serum albumin (Sigma), 10 ng/mL human basic fibroblast growth factor (bFGF; Sigma), 20 ng/mL epidermal growth factor (EGF; Sigma), 2% B27 Supplement (Invitrogen Corporation, Grand Island, NY) and antibiotic/antimycotic (Sigma). Cells were supplemented with fresh medium and growth factors twice weekly. At day 14, spheres >40 μm in diameter were collected using a cell strainer (BD), dissociated to single cells, and reseeded to evaluate self-renewal through the formation of a second generation of tumor spheres. In order to determinate putative CSC markers, an aliquot was analyzed by flow cytometry.

For differentiation assays, disaggregated spheres were seeded in RPMI 1640 medium with 10% FCS in adherent conditions.

In another set of experiments, OE33 cells were plated for the formation of esophageal spheres and then treated with celecoxib (10, 20, or 40 μM) for 7 days. Thus, we determined the size of spheres using Image J software at days 2 and 7 by measuring sphere diameter.

### Statistical analysis

Results are expressed as mean ± *SEM*. Student *t-*tests and analysis of variance followed by a Dunnet's test were used to calculate the statistical differences, and *p* < 0.05 was considered statistically significant.

## Results

### Enrichment in cancer stem cell markers by exposing cells to 5-FU

**Figure 1:**
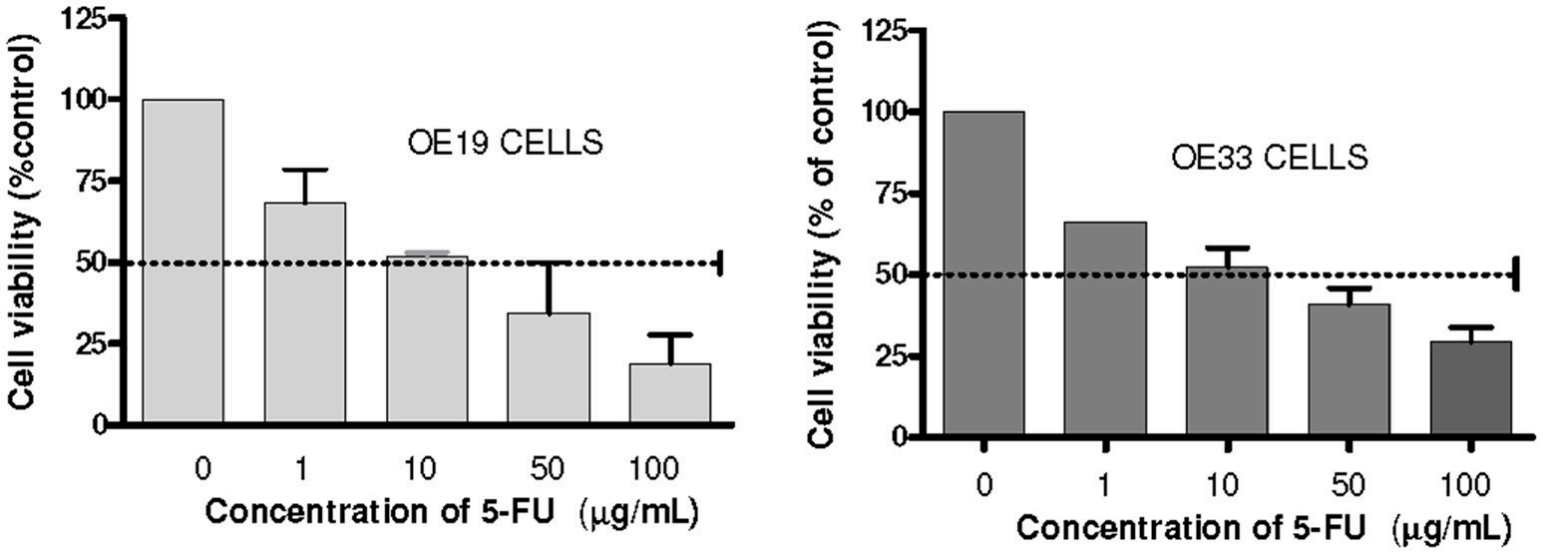
Analysis of cells chemosensitivity to 5-FU. Cell viability was measured using an MTT assay kit. The results are expressed as the mean ± *SEM* of cell viability percentage in 5-FU treated cells with respect to control cells of at least three independent experiments.

MTT assay was used to establish IC_50_-value for 5-FU in our cell lines. IC_50_ doses were obtained from the dose-response curves (GraphPad Prism 4.0, La Jolla, CA, USA) and were established at 10 μg/mL for OE33 cell line and 10.96 μg/mL for OE19 cell line (Figure 1). The fraction that survived 5-FU exposure was characterized for cancer stem phenotype and examined for enrichment in cancer stem markers.

**Figure 2:**
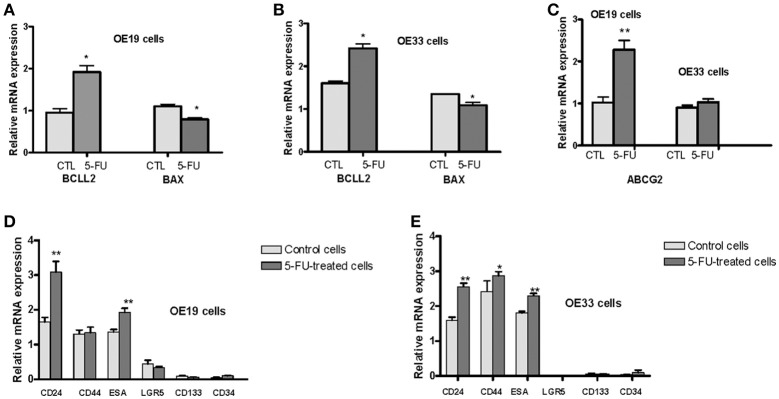
Analysis of mRNA levels of different markers related to stem phenotype. OE33 and OE19 EAC cell lines were exposed to 5-FU for 72 h. Relative gene expression analysis was developed in surviving cells vs. untreated cells. mRNA levels of the antiapoptotic gene *BCLL2* and the proapoptotic gene *BAX* in OE19 **(A)** and OE33 **(B)** cells. mRNA levels of the drug transporter *ABCG2* **(C)**. mRNA levels of CSC markers *CD24, CD34, CD44, CD133, ESA, LGR5* in OE19 **(D)** and OE33 **(E)** cells. Each bar represents the relative gene expression obtained after applying the following ratio: Normalized gene expression value/Kan-r gene value. Significant differences from the respective control values: ^*^p < 0.05; ^**^p < 0.01.

To characterize whether cells that survived 5-FU exposure were related to the cancer stem phenotype, we compared gene expression of genes involved in cell survival (*BAX* and *BCLL2*) and drug resistance (*ABCG2*) among cells surviving 5-FU exposure and control cells. Using the GeXP genetic analysis system, we determined mRNA expression of the *BAX* pro-apoptotic gene, the *BCLL2* antiapoptotic gene, and the *ABCG2* gene in the 5-FU-resistant fraction and in untreated cells. *BCLL2* expression was significantly increased in 5-FU treated cells (102% in OE19 cells, *p* = 0.001 and 51% in OE33 cells, *p* = 0.002) vs. untreated cells. In contrast, *BAX* gene expression was significantly reduced in treated cells (28% in OE19 cells, *p* = 0.002 and 19% in OE33 cells, *p* = 0.02; Figures 2A,B).

Drug resistance of CSCs is mediated in part by the ATP-binding cassette (ABC) drug transporters. Therefore, we examined *ABCG2* expression in these cells. Although levels of *ABCG2* mRNA were increased in 5-FU-treated OE19 cells (123% vs. control cells, *p* = 0.001), no difference in expression was observed in OE33 cells (Figure 2C).

Next, we determined mRNA expression levels of a variety of surface markers related to CSCs (*CD24, CD34, CD44, CD133, ESA*, and *LGR5*) in the 5-FU-resistant fraction and untreated cells. Expression of all analyzed markers was observed in both cancer cell lines with the exception of *LGR5*, which was not detected in the OE33 cell line. *CD24, ESA*, and *CD44* were the main CSC markers expressed. Cells surviving treatment with 5-FU exhibited a significant increase of *CD24* mRNA expression (87% in OE19 cells, *p* = 0.04 and 66% in OE33 cells, *p* = 0.001) and a moderate increase of *ESA* mRNA expression (40% in OE19 cells, *p* = 0.004 and 27% in OE33 cells, *p* = 0.005) compared with untreated cells. No statistically significant differences were observed when the mRNA expression of *CD34, CD133*, and *LGR5* was analyzed. The level of *CD44* mRNA was slightly increased (18%, *p* = 0.027) in 5-FU treated OE33 cells (Figures 2D,E).

### *CD24* and *ESA* protein expression in EAC cell lines

Given that identification of CSC is performed using cell surface markers, and based on the above results, we determined by immunofluorescence the specific cellular localization and by flow cytometry the levels of surface protein *CD24* expression and *ESA* in 5-FU treated cells and in untreated cells.

**Figure 3:**
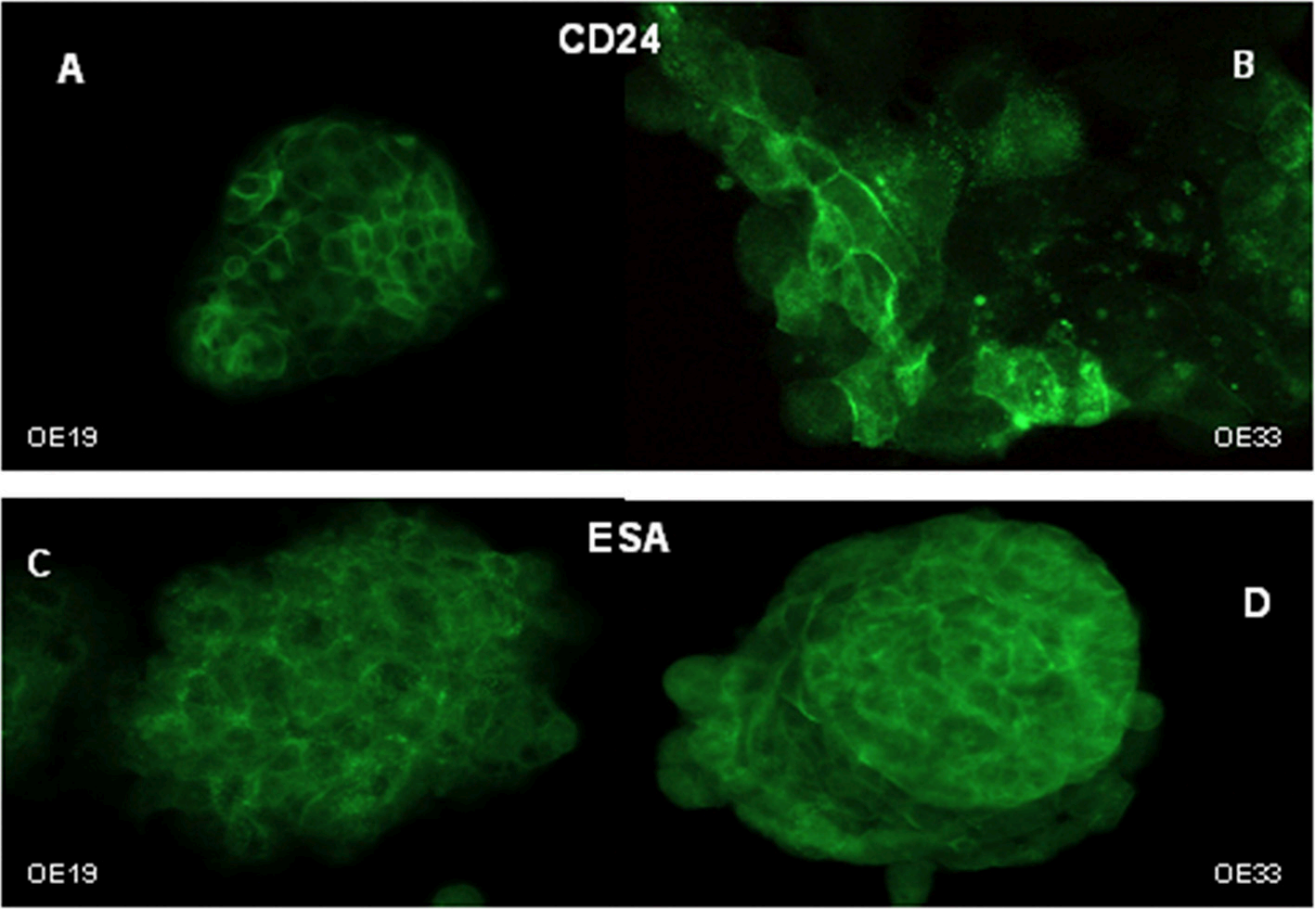
Cellular distribution of *CD24* and *ESA* markers. Immunofluorescent staining of *CD24* in OE19 **(A)** and OE33 **(B)** cells and *ESA* in OE19 **(C)** and OE33 **(D)** cell lines.

*CD24* staining intensity in esophageal cancer cells was very heterogeneous; that is, not all of the tumor cells displayed similar levels of protein expression (Figures 3A,B). The distribution of *CD24* protein was predominantly membranous in OE19 cells (Figure 3A), while membranous and cytoplasmic staining were observed in the OE33 cancer cell line (Figure 3B).

**Figure 4:**
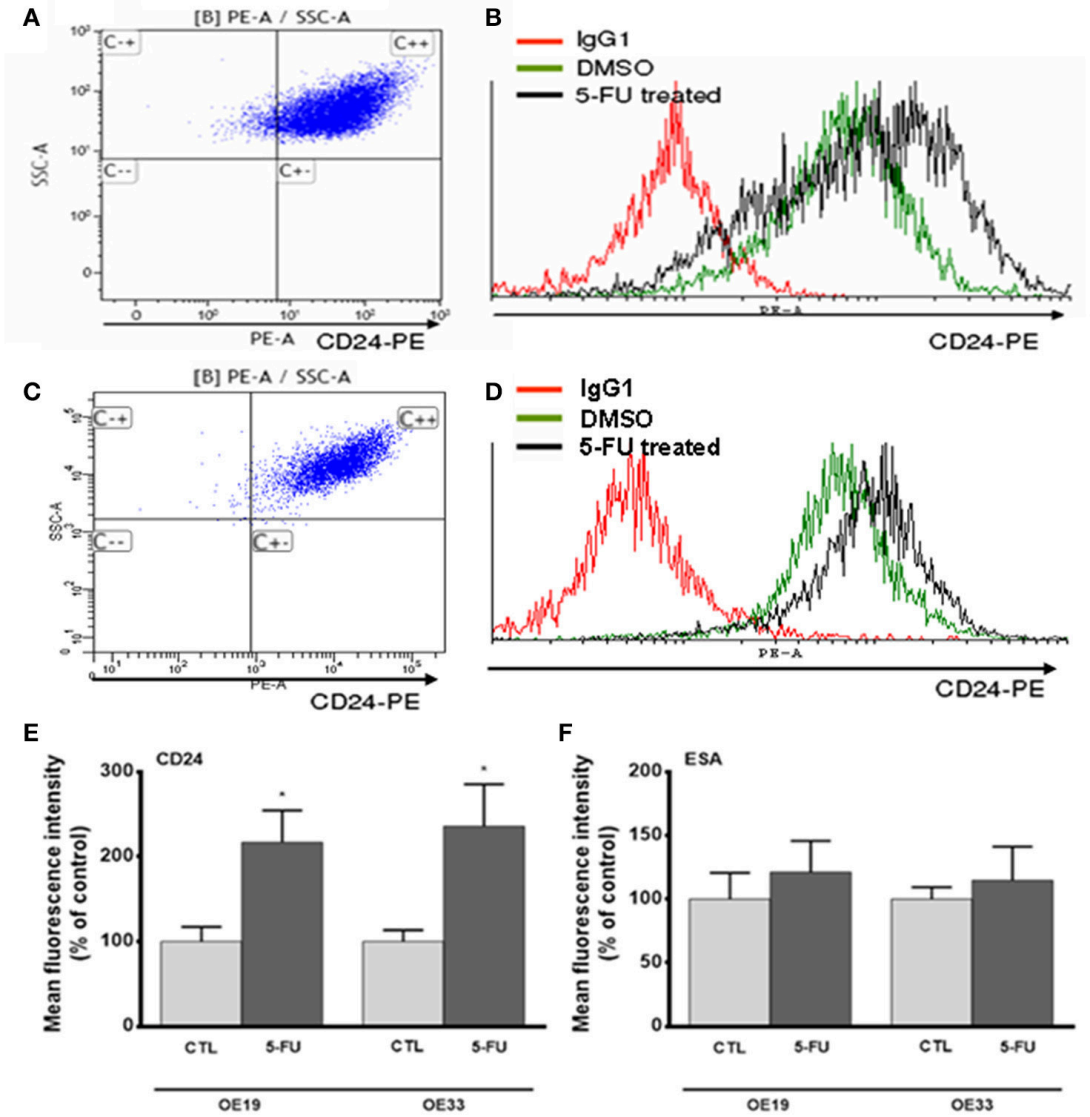
Analysis of *CD24* and *ESA* expression after 5-FU treatment. Representative image of flow cytometry dot plot of *CD24* expression in OE33 **(A)** and OE19 **(C)** tumor cells. Representative flow cytometry histograms displaying *CD24* expression in OE33 **(B)** and OE19 **(D)** cells treated with 5-FU (10 μg/mL) for 72 h (black line) or in cells treated with the vehicle alone (green line). Mean fluorescence intensity of *CD24* **(E)** and *ESA* **(F)** in 5-FU treated or in control cells. All data are expressed as the mean ± *SEM* of mean fluorescence units in treated cells relative to untreated cells of at least three independent experiments. Significant differences with respect to control cells: ^*^p < 0.05.

Flow cytometry revealed that the intensity of *CD24* expression, measured as mean fluorescence units (MFI) was significantly higher in 5-FU-treated cells than in control cells in both cell lines (OE19 cells *p* = 0.046; OE33 cells *p* = 0.035; Figures 4A–E).

*ESA* immunostaining revealed membranous and cytoplasmic staining in both cancer cell lines (Figures 3C,D), with apparently homogenous staining. No differences were observed in the *ESA* MFI between control and treated cells (Figure 4F).

### Sphere formation

**Figure 5:**
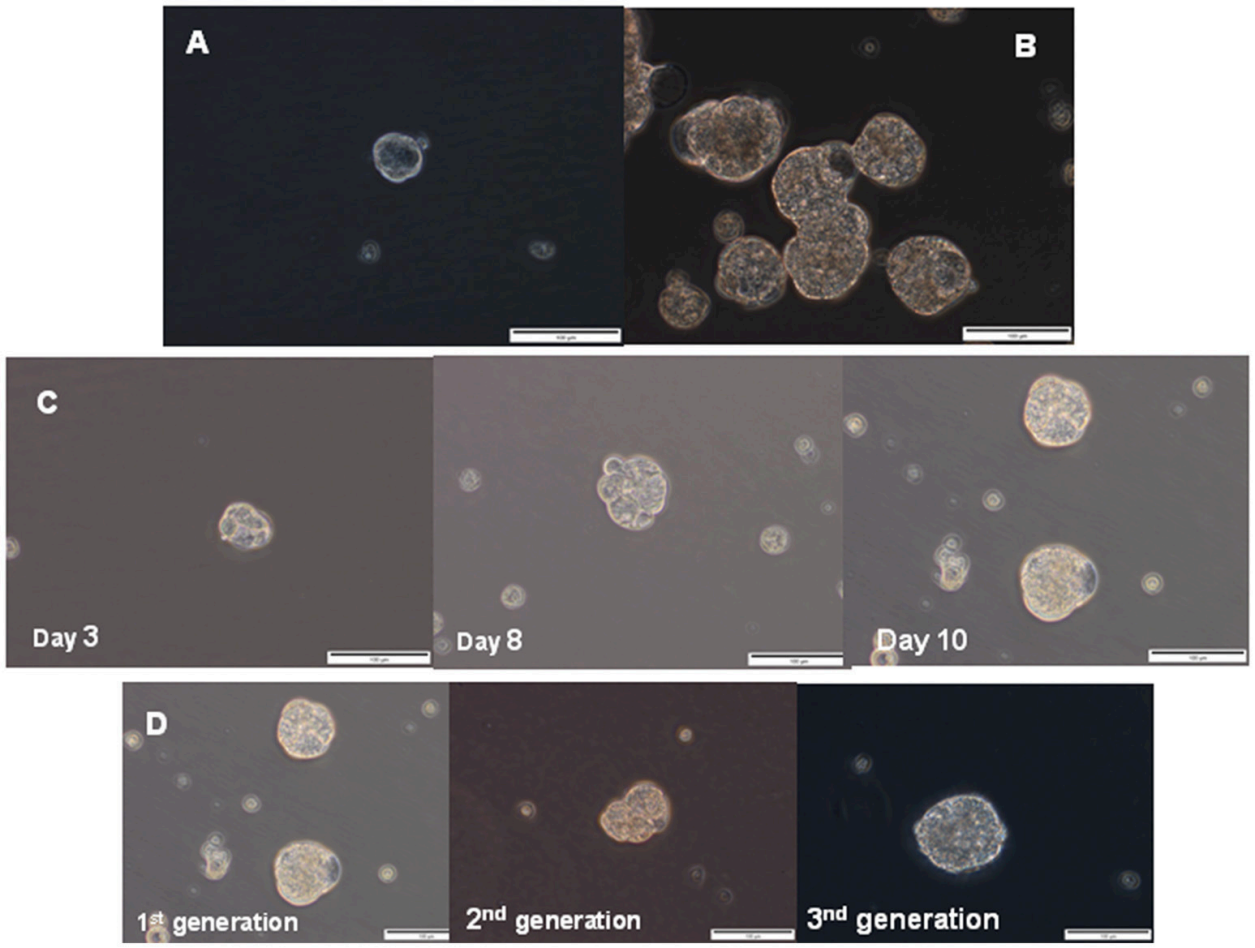
Sphere formation assay. Representative phase contrast photomicrographs of tumor spheres generated by suspension culture of OE19 **(A)** and OE33 **(B)** EAC cells after 14 days of culture. Representative images of first-generation spheres' growth, established from adherent cultures **(C)**. Representative images of the first-generation of spheres (corresponding to spheres 10 days after seeding, as previously shown in Figure 4C) and two successive generations of spheres **(D)**.

Sphere culture has been used as a method for enriching stem cells and evaluating the stem cell self-renewal process. Therefore, we investigated whether EAC cells were able to form spheres *in vitro* when plated in a serum-free media supplemented with growth factors and in anchorage-independent conditions, which allow stem cells to maintain an undifferentiated status (Singh et al., 2003; Cao et al., 2011; Chen et al., 2012). Both esophageal cancer cell lines had the capacity to form multiple spheres, although OE33 cells showed a greater ability to generate spheres than OE19 cells (Figures 5A,B). Spheres also increased in size over time (Figure 5C). After 14 days under these culture conditions, spheres derived from OE33 cells ranged from 80 to 100 μm in diameter and spheres derived from OE19 cells ranged from 50 to 60 μm in diameter.

#### Characterization of sphere culture

Esophageal cancer spheres displayed typical stem cell functionalities such as self-renewal, assessed as the spheres' capacity to produce second-generation spheres. Spheres derived from the OE33 cancer cell line had morphological characteristics similar to the first generation after they were passaged for at least three generations (Figure 5D).

**Figure 6:**
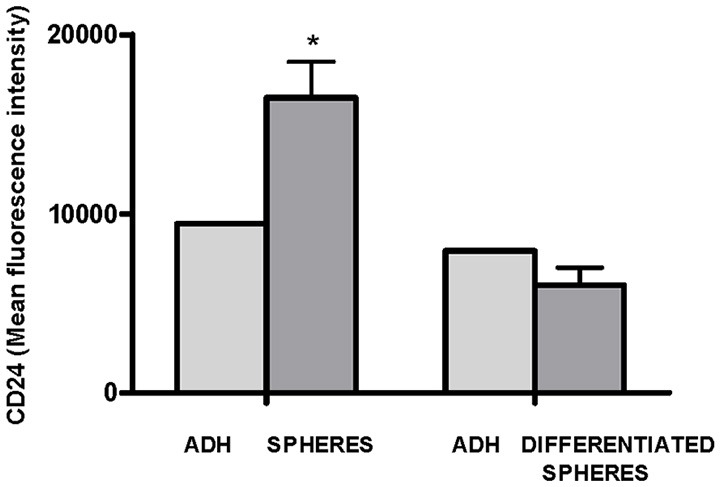
Flow cytometric analysis of *CD24* expression in adherent OE33 cells, OE33 spheres and differentiated spheres. All the results were expressed as mean ± *SEM* of mean fluorescence units of three independent experiments. ^*^p < 0.05 with respect to adherent cells.

Finally, we evaluated whether there were differences in the levels of *CD24* expression between cells from spheres and cells differentiated from spheres. A statistically significant higher MFI of *CD24* expression was observed in cells from spheres (75%, *p* = 0.025) than in the parental adherent (ADH) cells. When the cells from spheres were plated into adherent plastic plates with a culture medium supplemented with FBS and cultivated for 4 weeks, a lower MFI of *CD24* expression was observed, which reached levels similar to the levels observed in the parental cells (Figure 6).

### Role of celecoxib in *CD24* expression and tumor sphere formation

**Figure 7:**
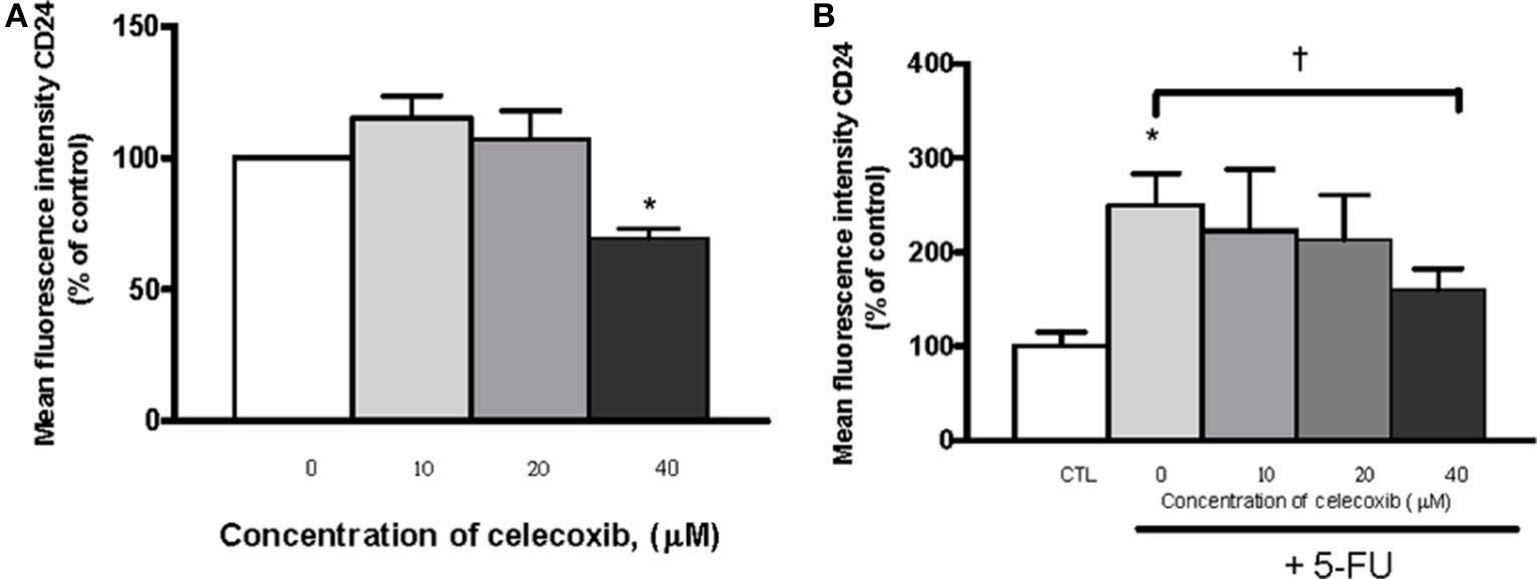
Effects of celecoxib and/or 5-FU on *CD24* expression. The effects of the treatment were expressed as mean fluorescence units of OE33 cells treated with different concentrations of celecoxib (0–40 μM) alone **(A)** or in combination with 5-FU (10 μg/mL) **(B)** with respect to control (untreated) cells. All the results were expressed as mean ± *SEM* of mean fluorescence units in treated cells relative to untreated cells of three independent experiments. ^*^p < 0.05 vs. control; ^†^*p* < 0.05 vs. 5-FU alone.

To assess the effects of celecoxib on *CD24* expression, OE33 cells were incubated for 72 h with celecoxib (10, 20, or 40 μM) alone or in combination with 5-FU (10 μg/mL), and *CD24* MFI was analized by flow cytometry. When cells were treated with 5-FU alone, *CD24* expression was increased with respect to untreated cells. However, when cells were co-treated with 5-FU and 40 μM celecoxib, we found that celecoxib significantly attenuated 5-FU-induced *CD24* expression (*p* = 0.017). Likewise, cells treated with 40 μM celecoxib alone also showed a reduction of *CD24* compared with the corresponding untreated controls (*p* = 0.034). No effect was observed at the other doses of celecoxib tested (Figures 7A,B).

**Figure 8:**
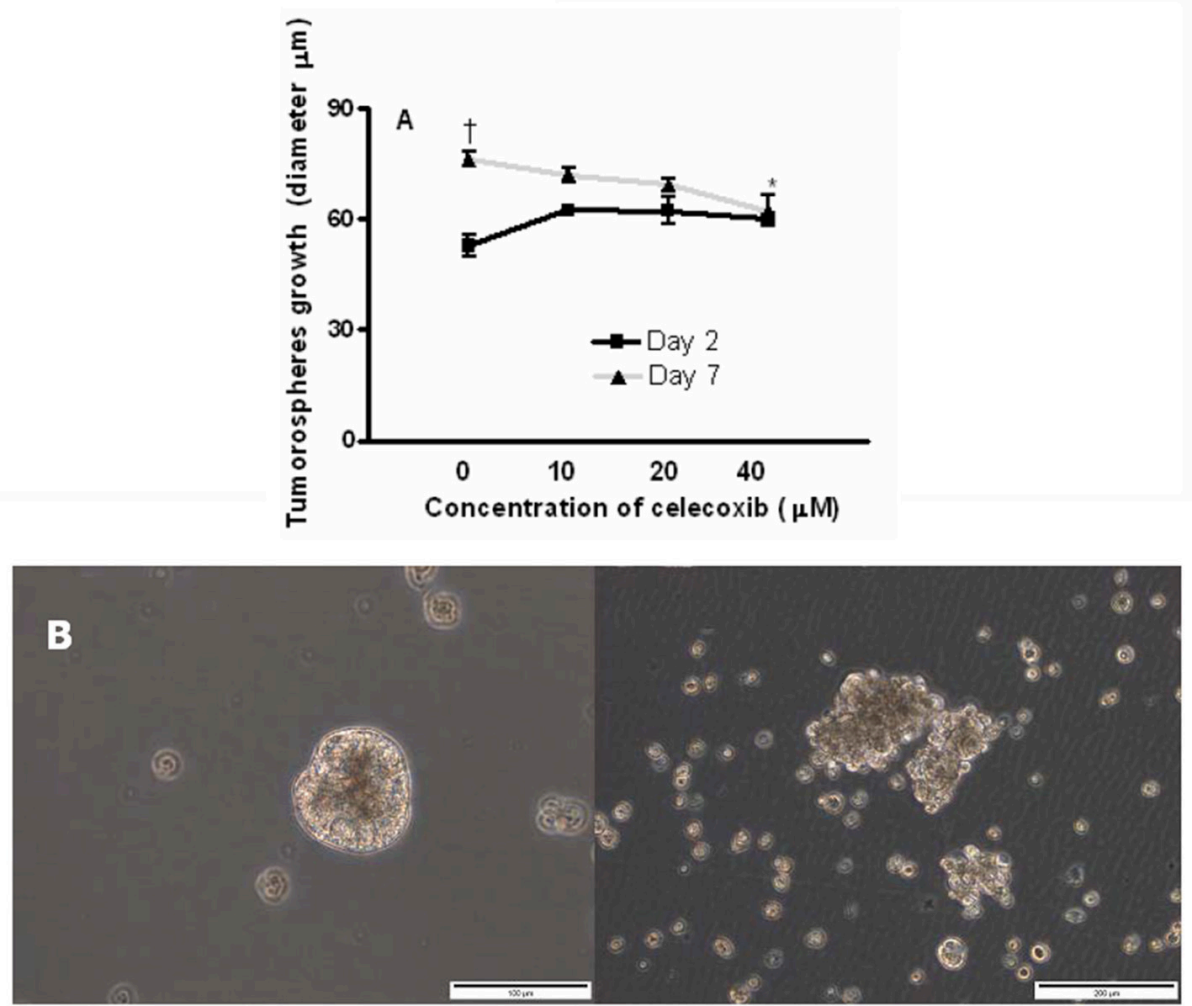
Effects of celecoxib on of esophageal tumor spheres' growth. Diameter of OE33 spheres after exposure to celecoxib (0–40 μM) for 2 or 7 days **(A)**. All the results were expressed as mean ± *SEM* of the diameter of spheres of three independent experiments. ^†^*p* < 0.05, diameter of untreated spheres at day 2 vs. diameter of untreated spheres at day 7; ^*^p < 0.05, diameter of spheres treated with celecoxib (40 μM) vs. untreated control at day 7. A representative photograph showing the disintegration of OE33 spheres after incubation with 40 μM celecoxib for 7 days (**B**, right) (200 × magnification).

To evaluate the effects of celecoxib on tumor sphere formation, OE33 cells were grown as tumor spheres and incubated with celecoxib (10, 20, or 40 μM) for 7 days. We monitored the size of spheres in the different experimental conditions (control and treatment) by measuring the diameter of the spheres with a well-defined spherical shape (Figure 8A). After 7 days of treatment, celecoxib (40 μM) significantly inhibited the growth of the spheres compared to untreated (control) cells (*p* = 0.01).

In some cases, we also observed that the treatment of spheres with celecoxib for 7 days resulted in the disintegration of these spheres compared with the corresponding controls (Figure 8B).

## Discussion

CSCs research might offer a new class of oncologic biomarkers that may improve the diagnosis, prognosis, treatment, and/or risk of progression in premalignant lesions.

The enrichment of CSCs after chemotherapy treatment has been observed in *in vitro* models, as well as in clinical practice. *In vitro* models have demonstrated that CSCs can be selected from the parental population of tumor cells by treating cells with chemotherapy drugs (Levina et al., 2008; Yu et al., 2009; Zhang et al., 2010; Abubaker et al., 2013; Canter et al., 2014). In the clinical practice, it has been reported that tumors from patients treated with chemotherapy exhibited a higher proportion of CSCs than tumors from untreated patients (Yu et al., 2007; Li et al., 2008). Therefore, our first objective was to investigate the presence of cell-surface markers related to the stem phenotype implicated in chemoresistance on esophageal cancer cells. 5-FU-treated OE19 cells showed elevated expression of *ABCG2*, and both tested cell lines exhibited increased resistance to apoptosis, properties consistent with stem cell phenotype. In our experiments we observed that treatment with 5-FU induced an increase in mRNA levels of CSC markers *CD24* and *ESA*, but only increased levels of *CD24* protein expression were observed, which could be probably due to post-transcriptional mechanisms.

*CD24* acts as a ligand for P-selectin and appears to contribute to the acceleration of tumor growth and metastases (Kristiansen et al., 2004b; Baumann et al., 2005; Burgos-Ojeda et al., 2015). In breast cancer, *CD24*-expressing cancer cells can disseminate more readily through their capacity to form thrombi with platelets or bind to endothelial cells in the bloodstream (Aigner et al., 1998). The loss of *CD24* function has also been related with induction of apoptosis and decreased rates of cell proliferation in several tumor cell lines (Smith et al., 2006).

In recent years, *CD24* overexpression has been correlated with shorter patient survival in breast cancer, ovarian cancer, non-small cell lung carcinomas, prostate tumors, colorectal cancer, and in esophageal squamous cell carcinoma (Kristiansen et al., 2002, 2003, 2004a; Choi et al., 2005; Sano et al., 2009; Majores et al., 2015; Wang et al., 2016). *CD24* expression has also been associated with bladder tumor recurrence (Liu et al., 2013). *CD24* upregulation has been shown during the progression of colorectal cancer and has been considered a potential target for early intervention in the prevention and treatment of colorectal cancer (Sagiv et al., 2006, 2008; Kraus et al., 2015). *CD24* is considered one of the surface markers associated with CSCs, mainly in the pancreas (Li et al., 2007). Heterogeneous staining/expression of cell-surface antigens has previously been documented and has been recognized in normal cells, in tumor cells, and also in long-established tumor cell lines (Edwards, 1985; Jungbluth et al., 2001; Al-Hajj et al., 2003). In our study, we observed higher *CD24* expression in cells that survived chemotherapy, as well as in OE33 sphere-forming cells compared with adherent cells. Moreover, we found that esophageal tumor cells exhibited heterogeneous *CD24* protein expression, suggesting the presence of different populations or cells with different differentiation states within the EAC cell culture. Although *CD2*4 is a membrane receptor, we observed membranous and cytoplasmic immunostaining in the present study. OE19 cells, a cell line obtained from a well-differentiated tumor, showed membranous staining; while OE33 cells, derived from a poorly differentiated adenocarcinoma, displayed strong cytoplasmic staining, and focal membranous expression. These results are in accordance with previous data obtained in biopsy specimens from colorectal, breast, prostate, ovarian, and gastric cancer, in which a higher cytosolic expression of the receptor has been associated with a more invasive phenotype and reduced patient survival (Kristiansen et al., 2002, 2003, 2004a; Choi et al., 2005; Weichert et al., 2005; Chou et al., 2007; Sano et al., 2009; Majores et al., 2015). The biological significance of the shift from the normal membranous expression to the cytoplasmic location of *CD24* is still unclear, and might be due to accumulation in membrane vesicles released into the cytosol.

The model of tumor sphere formation has been shown to closely mimic phenotype characteristics of *in vivo* solid tumors, and to allow *in vitro* propagation of CSCs (Weiswald et al., 2010). In colon cancer, primary tumors exhibited a correlation between tumor aggressiveness and the ability of cancer cells to form numerous colonospheres (Weiswald et al., 2009). In esophageal cancer, our results showed that both EAC cell lines formed spheres, although OE33 cells displayed a greater capacity to form spheres.

Most oncological studies with NSAIDs have principally focused on their potential chemopreventive effect; however, celecoxib has also demonstrated antineoplastic activity against different types of cancer *in vitro* and also in animal models (Masferrer et al., 2000; Dannenberg and Subbaramaiah, 2003; Koehne and Dubois, 2004; Riva et al., 2016). It has also been suggested that celecoxib might have clinical potential as an adjuvant therapy in patients with EAC (Tuynman et al., 2005); however, the molecular mechanisms and cellular targets through which celecoxib exert its anti-tumor effects are not well-known. On one hand, there is clear evidence that *COX-2* inhibition downregulates important proteins involved in cancer progression and dissemination, and many of the *COX-2*- regulated genes may determine tumor chemosensitivity (Dannenberg and Subbaramaiah, 2003; Tuynman et al., 2005; Tsujii, 2013). Moreover, cultured *COX-2*-expressing cells from colorectal cancer tissues exhibited several characteristics related to stem cells, such as spheroid-forming ability, chemoresistance, and cell cycle-arrest (Tsujii, 2013). On the other hand, several reports indicate that anti-tumor activity of celecoxib is independent of *COX-2* inhibition (Schönthal, 2006; Ryan et al., 2008). Thus, in colon carcinoma cell lines, both celecoxib (a *COX-2* selective inhibitor) and SC-560 (a *COX-1* selective inhibitor) had effects on cell survival, apoptosis, and cell cycle arrest independently of the cells' *COX-2* expression (Grösch et al., 2001).

Our study indicates that celecoxib decreased *CD24* expression both alone and in combination with 5-FU; that is, the increased *CD24* expression observed after 5-FU administration was attenuated when 5-FU was combined with celecoxib. Since treatment with 40 μM celecoxib had previously shown to decrease cell viability of OE33 cells (Tuynman et al., 2005), we cannot discard that the reduced CD24 levels observed are due to the cytotoxic effects of the treatment. Previous reports also showed that increased *CD24* expression reverted to a normal level after short (72 h) and long (6 months) exposure to celecoxib in colon cancer (Sagiv et al., 2006). It should be noticed that, as in the present study, those effects were also observed at higher concentrations than those achieved in human serum following standard anti-inflammatory dosing (Niederberger et al., 2001). In OE33 sphere cultures, celecoxib was unable to prevent the initiation of tumor spheres at all of the tested doses; however, celecoxib disaggregated esophageal cancer cell spheres after 7 days of treatment, suggesting a potential role of celecoxib in the cancer stem model. Nevertheless, a possible limitation of our study lies in the number of cell lines evaluated, due that the population of cancer cells within a tumor is quite diverse and cell lines evaluated in this study could not be sufficiently representative of the heterogeneity of the primary tumor.

Taken together, these findings provide evidences that esophageal cancer cell lines are heterogeneous with regard to *CD24* expression. Our observations demonstrate that administration of the chemotherapeutic 5-FU leads to an increase in *CD24* marker expression in esophageal adenocarcinoma cell lines, which may be related to the stem cell phenotype, as suggested by its increased expression in esophageal cancer spheres. Our results also suggest that *CD24* might have prognostic and therapeutic implications for the treatment of esophageal cancer, and that celecoxib could be evaluated as a possible candidate for combined chemotherapy with 5-FU in the treatment of esophageal cancer.

## Author contributions

PJ was involved in the conception and design of the study, performed most experiments, and wrote the paper. EC was involved in writing and editing the manuscript. PJ, MA, MS, ES, and MG performed the experiments. TS was involved in the revision of the manuscript. AL was involved in the revision of the work and provided vital reagents and analytical tools.

### Conflict of interest statement

The authors declare that the research was conducted in the absence of any commercial or financial relationships that could be construed as a potential conflict of interest.
